# Cognitive performance in young adult women with a history of premature adrenarche

**DOI:** 10.1038/s41390-024-03380-4

**Published:** 2024-07-05

**Authors:** Jussi Tennilä, Liisa Muukkonen, Pauliina Utriainen, Raimo Voutilainen, Jarmo Jääskeläinen, Jani Liimatta

**Affiliations:** 1 Kuopio Pediatric Research Unit, University of Eastern Finland and Kuopio University Hospital, 70211 Kuopio, Finland; 2 Coronaria Psykiatria, 80100 Joensuu, Finland; 3 Research Program for Clinical and Molecular Metabolism, Faculty of Medicine, University of Helsinki, 00014 Helsinki, Finland; 4 Pediatric Research Center, Children’s Hospital, Helsinki University Hospital, 00029 Helsinki, Finland; 5 Department of BioMedical Research, University of Bern, 3012 Bern, Switzerland

## Abstract

**Background:**

Girls with premature adrenarche (PA) mature earlier than peers and have been found to have greater symptom accounts reflecting anxiety compared to peers. It is not known, however, whether PA effects cognitive development. This longitudinal case-control cohort study aimed: (1) To investigate whether a history of PA leads to measurable changes in adulthood cognitive performance, and (2) to assess whether findings characteristic of PA girls predict adulthood cognitive performance.

**Methods:**

Twenty-seven girls with PA and 27 age-matched control girls were examined and followed from mid-childhood (mean age 7.2 years) until early adult age (18.5 years). Wechsler Adult Intelligence Scale, Fourth Edition scores were used as main outcome measure.

**Results:**

Allostatic load (AL) scores, which compile multisystem variables to reflect the overall wear and tear of the body from increased and prolonged stress, were higher in the PA group in both prepuberty and adulthood, but there were no differences in WAIS-IV results between the groups (full-scale IQ 92.7 vs. 97.5, p 0.376; no differences in separate indexes). Childhood androgen levels, glucose metabolism biomarkers, and AL scores failed to predict adulthood cognitive performance outcomes.

**Conclusion:**

The study suggests that PA does not predispose to adverse adulthood outcomes of cognitive development.

**Impact:**

- The study suggests that a history of premature adrenarche (PA) does not affect cognitive performance in adult age.
- Childhood androgen levels and biomarkers of glucose metabolism failed to predict adulthood cognitive outcomes in this study.
- Allostatic load scores were elevated in the PA group both in childhood and adulthood but did not predict adulthood cognitive outcomes.

## Introduction

Adrenarche refers to the maturation of the innermost layer of the adrenal cortex leading to increased adrenal androgen secretion in mid-childhood. In premature adrenarche (PA) this maturation leads to clinical signs of adrenal androgen action (comedones/acne, oily hair, and skin, axillary/pubic hair, adult type body odor) before the age of 8 years in girls and 9 years in boys.^1^ Girls with PA experience menarche earlier than others, and have increased height, weight, androgen levels, and insulin resistance (IR) compared to peers.^1^ For most individuals with prior PA, these changes tend to attenuate by adult age,^1–3^ but the potential long-term outcomes of PA are still debated.^1,3^

Hyperandrogenism in PA is caused by increased secretion of adrenal androgens, namely 11-oxygenated androgens, androstenedione, dehydroepiandrosterone (DHEA) and its sulphate conjugate DHEAS (DHEA and DHEAS jointly henceforth termed DHEA(S)). DHEA(S) are neuroactive steroids with various effects in the brain.^4^ Findings from animal studies suggest that they exert neuroprotective effects, promote neurite growth and neurogenesis, affect apoptosis, protect against catabolic effects of glucocorticoids, and have an anti-inflammatory function.^4^ Because of the timing of adrenarche and related increases in DHEA(S) right before transitioning from childhood to adolescence, it has been proposed that the function of adrenarche is to help preadolescents adapt and rewire in the face of a myriad of ever more complex and demanding social interactions.^5^ Concurrently, results of imaging studies suggest that DHEA(S) in adrenarche might indeed play a critical role in the developing brain, influencing brain structure and connectivity in several key brain regions.^6^ These changes in turn, when appropriately timed, are associated with positive changes in executive function^7^ and other cognitive domains.^5,6,8^ For example, higher DHEA levels in adolescence have been found to correlate negatively with structural covariance in volume between amygdala and right occipital regions, and lesser covariance between the two was associated with better visual awareness and processing speed.^7^ Similarly, DHEA-related higher insular-hippocampal covariance has been linked with better overall working memory.^8^

The continuing growth and reorganization of the preadolescent brain, however, makes it a moving target for hormonal effects, so that variation in the timing of hormone exposure in adrenarche^9^ and gonadarche^10^ may lead to changes in neurodevelopmental trajectories, and, thereby, to variation in adulthood outcomes. In addition to possible direct impacts of steroid hormones and insulin resistance on brain structure and function, the timing of maturational changes can influence long-term neurodevelopmental outcomes indirectly—through stress related mechanisms.^11^ Earlier maturation relative to peers can make the transition from childhood to adolescence especially challenging and confusing.^11^ Indeed, earlier menarche relative to peers, as a proxy for earlier maturation, has been linked with adverse neuropsychological consequences,^11^ and PA children going through menarche earlier than peers were previously found to have increased prevalence of oppositional defiance disorder and greater symptom accounts reflecting anxiety compared to peers.^12^ Such added stress may further impact the divergence of developmental paths.^9^ Over time, prolonged stress may also lead to maladaptive physiological, immunological, and metabolic changes. These multisystem changes, jointly termed “allostatic load” (AL), reflect the overall wear and tear of the body, and have been shown to negatively correlate with cognitive performance in both young and older age groups.^13^ Lastly, insulin resistance even without hyperglycemia, commonly found in PA girls, who tend to be overweight,^1^ has also been implicated in decreased cognitive performance.^14^

PA might, then, impact long-term cognitive outcomes in two ways. First, premature and excessive adrenal androgen exposure and decreased insulin sensitivity might exert direct organizational effects on the developing brain. Second, as PA individuals mature slightly earlier than their peers, they are susceptible to increased psychosocial stress, which, independently or in conjunction with hormone effects, may lead to altering of cognitive outcomes in adulthood. If this is true, then adult PA individuals could show alteration in cognitive performance compared to controls, and such alteration should be partially attributable to childhood DHEAS(S) and/or glucose metabolism. In addition, one should expect to see a persisting elevation in AL scores of the PA group from childhood to adulthood and a meaningful effect size for those scores on cognitive performance outcomes. Cognitive performance of PA individuals, however, has only been studied in children with mixed results.^15–17^ Investigation of long-term cognitive consequences of PA is warranted because general cognitive ability, i.e., intelligence, predicts a wide range of socio-economical and health related outcomes, including all-cause mortality.^18^

In this study we followed a cohort of PA and age-matched control girls from preadolescence until early adulthood with the primary aim of gaining insight into whether a history of PA leads to measurable changes in cognitive outcomes in adulthood. In addition, we searched for childhood factors that would predict adulthood cognitive performance.

## Methods

### Participants

Altogether 63 PA and 80 control girls of Caucasian ethnicity were (originally) recruited in the Northern Savo region in Finland between 2004 and 2006. PA girls presented with at least one clinical sign of adrenarche (adult-type body odor, oily skin, comedones/acne, development of axillary and/or pubic hair) before the age of 8 years. Exclusion criteria included any other endocrine disease and long-term medication.^19^ In addition, we later invited all women (with the same exclusion criteria) who had been evaluated in our pediatric outpatient clinic for PA during 2000–2008, of which seven were willing to participate. The control group was obtained from the Finnish population registry and consisted of healthy, randomly selected, age-matched girls from the same area as the PA group. Altogether 30 PA subjects and 42 controls attended the adulthood follow-up (mean age 18.5 years), of which 28 PA and 29 controls agreed to perform the Wechsler Adult Intelligence Scale, Fourth Edition (WAIS-IV) assessment.^20^ Reasons for not agreeing are unknown. One PA subject and two controls were excluded due to birth before 35 weeks of gestational age. Thus, final group sizes of the current study were 27 PA and 27 control women (Supplemental Fig. 1), with a mean follow-up time of 11 years and 4 months.

Reasons for not attending the adulthood follow-up are unknown, but the non-attendees had similar birth weight, prepubertal weight, height, body mass index (BMI), DHEA(S) concentrations and prevalence of pubarche as the attendees. A letter with a questionnaire was sent to the 40 PA and 38 controls who did not attend, asking them to describe their educational status at 18 years of age and to report their comprehensive school education diploma mean grade and parental educational levels. Twelve PA and 11 controls responded. We detected no specific factor distinguishing the non-attendees from others (See Supplemental Material, Figs. 2, 3).

Informed consents were received according to the Declaration of Helsinki, and the study protocol was approved by the Research Ethics Committee of Hospital District of Northern Savo, Finland.

### Anthropometric measurements

Acquisition of birth and childhood anthropometric measurements have been reported previously.^19^ Birth weight and length were converted to standard deviation scores (SDS) based on sex and gestational age specific Finnish growth charts.^21^ BMI was calculated as (weight (kg)/height (m)^2^) and prepubertal height and BMI were converted to SDS according to recent Finnish growth references.^22^ ISO-BMI was translated from prepubertal BMI SDS, and it represents BMI that corresponds to BMI used for adults.^22^ At adult age, height was measured with a calibrated Harpenden stadiometer (Holtain Ltd., Crymych, United Kingdom). Weight was measured with a calibrated electronic scale and head circumference as the maximum fronto-occipital circumference. All were recorded to the nearest 0.1 cm/kg. Blood pressure was measured with a standard sphygmomanometer from the left arm in sitting position after a 30-min rest and recorded as the average of three repeated measurements. Background information concerning family members, age at menarche, illnesses, medication, and alcohol use were gathered with a questionnaire form.

### Biochemical analyses

All blood samples were drawn after an overnight fast between 07 am and 10 am, and stored in −80 °C until assayed.

The following methods were used in prepuberty. DHEA and DHEAS were measured with RIA (Alpha Diagnostics International, SA, TX), cortisol with an electrochemiluminescence immunoassay (ECLIA) (Roche Diagnostics International AG, Rotkreuz, Switzerland), glucose with an oxidase method (Clarke Electrode, Tarrytown, NY), insulin with a time-resolved fluoroimmunoassay by AutoDelfia (Perking Elmer, Turku, Finland), HbA1c with liquid chromatography (Tosoh Co., Tokyo, Japan), IGF-1 with immunochemiluminometric assay (IMMULITE 2000 analyzer; Siemens Healthcare GmbH, Erlangen, Germany), and cholesterol and triglycerides using enzymatic methods (Thermo Electron Co, Vantaa, Finland).

The following methods were used in adulthood. DHEAS was measured using RIA (Alpha Diagnostics International, SA, TX), cortisol with ECLIA (Roche Diagnostics International AG, Rotkreuz, Switzerland), IGF-1 with ELISA kit (Mediagnost, Reutlingen, Germany), glucose with the hexokinase method using a COBAS-analyzer (Hitachi High Technology Co., Tokyo, Japan), insulin with ECLIA (Roche Diagnostics International AG, Rotkreuz, Switzerland), HbA1c with turbidimetric inhibition immunoassay and a COBAS-analyzer (Hitachi High Technology Co., Tokyo, Japan), total cholesterol with calorimetric enzymatic assay, high-density lipoprotein cholesterol (HDL-C) with homogenous enzymatic calorimetric assay and triglycerides with enzymatic calorimetric test (all with COBAS-analyzer; Hitachi High Technology, Tokyo, Japan). The following formula was used for calculating homeostatic model assessment for IR (HOMA-IR), (fasting glucose [mg/dL] × fasting insulin [mU/L])/405.

### Allostatic load

A total of 12 biomarkers at childhood and again at adult age were used for calculations of AL.^23^ The following subsystems and corresponding biomarkers were examined: neuroendocrine system with DHEAS and cortisol; immunological system with IGF-1; metabolic system with BMI, glucose, insulin, HbA1c, total cholesterol, HDL-C, and triglycerides; and cardiovascular system with systolic and diastolic blood pressure (BP). For each biomarker, the relevant clinical cut-off reference ranges were identified, and a dichotomous variable was created indicating whether each particular value was within reference ranges considered normal or low risk (coded “0”), or outside of said ranges, thereby considered high risk (coded “1”). These were then summed to create AL composite scores. In addition, to see if a different approach would change the results, we calculated z-scores for each biomarker with the following formula: (observed value—sample mean)/sample standard deviation. For more detailed information on these AL calculations, reference ranges used for each variable, and handling of the missing variables, see Supplemental material, Table 1. (21).

### Cognitive performance

Cognitive performance was assessed with the WAIS-IV assessment, and performed by a trained psychologist, who was unaware of which study group the participants belonged to. WAIS-IV consists of four indexes: verbal comprehension (VCI), perceptual reasoning (PRI), working memory (WMI) and processing speed (PSI). These are summed to create the full-scale IQ (FSIQ). The Finnish version of WAIS-IV has been standardized using a random sample of Finnish-speaking adults (*n* = 657, age range between 16 and 92 years). FSIQ and the four indexes (VCI, PRI, WMI, PSI) have a mean of 100 and SD of 15. Descriptive classifications of WAIS-IV FSIQ and all subtest results were defined as follows: extremely low, <69; borderline, 70–79; low average, 80–89; average, 90–109; high average, 110–119; superior, 120–129; very superior, >130.^24^

We also received final comprehensive school education diplomas from 23 PA and 20 controls. Three individuals, all in PA group, were ineligible for grade comparisons because they had received additional support, and possibly modified testing and evaluation principles. In Finland, children complete comprehensive school education at approximately 15–16 years of age. The Finnish school system grades individuals on a 7-ladder scale from 4 to 10, with one failed grade ( = “4”). Music, sports, arts, and handicraft were not included when calculating mean grades. In addition to calculating overall mean grade, language skills (Finnish, English, and Swedish), sciences (math, physics, chemistry, geology, health science, and biology), and social sciences (history, religion, and political science) were separately evaluated.

### Statistical analysis

All data were analysed with the IBM SPSS Statistics software version 27.0 (IBM Corp., Armonk, NY). Depending on distributions, continuous variables are expressed as mean (SD) or median (interquartile range) and analysed with the t-test or Mann–Whitney U test. Categorical variables are expressed as n (%) and analysed with either the chi-square or Fischer´s exact test. Correlations express the results from the Pearson or Spearman correlation tests. One-way ANCOVA was used for covariate adjustments. Linear regression models were used to examine predicting factors for adulthood outcomes and the following were used as dependent variables: FSIQ, VCI, PRI, WMI, PSI, and comprehensive school education mean grade. First, the childhood and adulthood AL composite scores, means of summed z-scores, and menarcheal age were separately used as independent variables. Second, we created two models to investigate the effects of specific childhood biochemical markers. Model 1 consists of childhood DHEAS, DHEA, and cortisol concentrations, while fasting plasma-glucose, HbA1c, and IGF-1 constitute model 2. The linear regression models were controlled for birth weight SDS, gestational age, and current illness at adult age. Analyses for effects on mean grade were also controlled for different schools attended. The models were tested for normality, linearity, homoscedasticity, and absence of multicollinearity. Case-wise deletion was adopted to handle missing variables. A *p* < 0.05 was considered significant.

## Results

One PA and one control woman were born preterm, yet both after week 35 of gestational age. The PA women had lower birth weight compared to controls (Table 1). At prepubertal age, the PA girls were taller and heavier, and had higher DHEA, DHEAS, glucose, insulin, and IGF-1 concentrations when compared to control girls (Table 1), and approximately half of the PA girls (52%) had reached pubarche. PA women had their menarche earlier than controls (Table 1).

**Table 1:** The participants’ background information from birth, childhood, and adult age.

	Control, *n* = 27	PA, *n* = 27	*p*
At birth			
Gestational age, weeks	40.4 (39.3–41.6)	40.1 (38.6–41.0)	0.288
Preterm, *n*(%)^a^			
Yes	1 (3.7)	1 (4.3)	>0.999
No	26 (96.3)	22 (95.7)	
Birth length, cm	50.8 (1.9)	49.6 (2.2)	**0.032**
Birth length SDS	0.40 (0.85)	−0.20 (1.10)	**0.030**
Birth weight, g	3730 (473)	3430 (635)	0.060
Birth weight SDS	0.08 (−0.33 − 0.89)	−0.32 (−0.80 − 0.29)	**0.032**
At prepuberty			
Age, years	7.2 (0.8)	7.3 (1.1)	0.502
Height, cm	123.4 (5.6)	130.0 (9.0)	**0.002**
Height, SDS	−0.20 (0.75)	0.90 (0.99)	**<0.001**
Weight, kg	24.7 (21.7–28.8)	30.2 (26.0–37.7)	**0.003**
BMI, SDS	0.20 (−0.67–0.70)	0.45 (−0.44–2.30)	0.155
Siblings^b^			
Yes	22 (84.6)	19 (82.6)	>0.999
No	4 (15.4)	4 (17.4)	
Pubarche			
Yes	0 (0)	14 (51.9)	**<0.001**
No	27 (100)	13 (48.1)	
DHEA, nmol/l	4.40 (2.38–5.93)	6.50 (4.60–11.83)	**0.003**
*Allostatic load components*			
DHEAS, umol/l	0.75 (0.50–1.33)	2.05 (1.10–3.92)	**<0.001**
Cortisol, nmol/l	241 (192–303)	190 (150–383)	0.355
IGF-1, nmol/l	18.3 (5.1)	25.3 (7.5)	**<0.001**
ISO-BMI	21.3 (19.1–23.0)	22.5 (19.8–31.6)	0.115
fP-glucose, mmol/l	4.8 (0.3)	5.0 (0.3)	**0.006**
Insulin, pmol/l	24.3 (20.0–30.4)	42.4 (22.2–57.1)	**0.008**
HbA1c, mmol/mol	33.7 (2.8)	34.1 (2.8)	0.707
Total cholesterol, mmol/l	4.3 (0.8)	4.3 (0.7)	0.914
HDL-C, mmol/l	1.6 (0.4)	1.4 (0.3)	0.211
Triglycerides, mmol/l	0.54 (0.43–0.68)	0.60 (0.48–0.74)	0.315
Systolic BP, mmHg	98.0 (9.1)	106.7 (9.7)	**0.003**
Diastolic BP, mmHg	59.3 (8.9)	65.6 (10.5)	**0.038**
At adult age			
Age, years	18.2 (0.6)	18.9 (1.9)	0.809
Age at menarche, years	13.0 (12.0–14.0)	11.5 (11.0–12.0)	**<0.001**
Weight, kg	59.8 (54.9–65.2)	62.6 (58.2–81.8)	**0.046**
Height, cm	165 (5)	167 (7)	0.130
HOMA-IR	1.96 (1.44–2.39)	2.44 (1.58–3.74)	**0.019**
Ethinyl estradiol use			
Yes	9 (33.3)	13 (48.1)	0.403
Anxiety or depression			
Yes	1 (3.7)	4 (15.4)	0.192
Alcohol use, times per week, *n*(%)			
Less than once per month	6 (24)	7 (37)	0.299
Once per month	10 (40)	8 (42)	
2–4 times per month	9 (36)	4 (21)	
2–4 times per week	0 (0)	0 (0)	
*Allostatic load components*			
DHEAS, umol/l	5.5 (3.3–8.0)	7.1 (4.3–9.5)	**0.045/0.004**^c^
Cortisol, nmol/l	521 (442–694)	628 (440–846)	0.246/0.284^c^
IGF-1, nmol/l	51.5 (11.3)	53.9 (15.1)	0.516
BMI	21.6 (19.6–24.0)	22.8 (21.0–28.7)	0.130
fP-glucose, mmol/l	5.1 (5.0–5.2)	5.2 (5.0–5.8)	0.066
Insulin, pmol/l	54.0 (37.4–62.9)	64.2 (42.6–92.0)	**0.029**
HbA1c, mmol/mol	31.8 (2.4)	33.9 (2.3)	**0.004**
Total cholesterol, mmol/l	4.0 (3.6–4.9)	4.1 (3.7–4.5)	0.869
HDL-C, mmol/l	1.8 (0.4)	1.6 (0.4)	0.235
Triglycerides, mmol/l	0.7 (0.7–0.8)	0.8 (0.6–1.0)	0.452
Systolic BP, mmHg	116 (10)	122 (15)	0.103
Diastolic BP, mmHg	70 (6)	72 (8)	0.544

Continuous variables expressed as mean (SD) or median (interquartile range) and analyzed with either the t-test or the Mann–Whitney u-test depending on the distribution. Categorical variables expressed as n (%) and analyzed with either the chi-square t-test or Fischer´s exact test. For allostatic load components the group sizes are 20 PA and 25 controls.

*BMI* body-mass index, *DHEA* dehydroepiandrosterone, *DHEAS* dehydroepiandrosterone sulfate, *IGF-1* insulin-like growth factor -1, *HbA1c* hemoglobin A1c, *HDL-C* high-density lipoprotein cholesterol, *BP* blood pressure, *HOMA-IR* homeostatic model assessment for insulin resistance, *SDS* standard deviation score.

^a^n(Control) 27, n(PA) 23.

^b^n(Control) 26, n(PA) 23.

^c^after ethinyl estradiol use adjustment.

Statistically significant *p*-values are in bold.

At adult age, ten (37.0%) PA and eight (29.7%) control women reported having been diagnosed with a long-term illness (including migraine (2 PA, 2 controls), hypothyroidism (1 PA), anxiety or bulimia (1 PA, 1 control), depression or sleep disorders (3 PA), allergic rhinitis (2 PA, 2 controls), asthma (1 PA, 1 control), rheumatoid arthritis (1 control) and familial hypercholesterolemia (1 control)). Prevalence of a history of either a depression or anxiety diagnosis was statistically comparable between the groups (Table 1), and there were no significant differences between the groups in the use of ethinyl estradiol contraception (Table 1), other regular medication (22.2% in PA and 14.8% in controls, *p* = 0.484) or selective serotonin re-uptake inhibitors (11.1% in PA and 3.7% in controls; *p* = 0.351). The PA women had similar BMI but higher insulin, HbA1c, HOMA-IR, and DHEAS than the controls. The PA group had higher mean AL composite score and the sum of z-scores at both prepubertal and adulthood examinations, which were primarily due to higher DHEAS in prepuberty and higher insulin concentrations in adulthood (Table 2).

**Table 2:** Allostatic load scores in childhood and adulthood for the 20 women with a history of premature adrenarche (PA) and 25 controls.

		Control, *n* = 25 Present, *n* (%)	PA, *n* = 20 Present, *n* (%)
**Childhood**			
Neuroendocrine	DHEAS	**8 (32)**	**15 (75)**
	Cortisol	2 (8)	1 (5)
Immunological	IGF-1	0 (0)	1 (5)
Metabolic	ISO-BMI	6 (24)	11 (55)
	fP-gluc	0 (0)	0 (0)
	Insulin	0(0)	2 (10)
	HbA1c	1 (4)	1 (5)
	Cholesterol	5 (20)	5 (25)
	HDL-C	4 (16)	5 (25)
	Triglycerides	1 (4)	1 (5)
Cardiovascular	sBP	4 (17)	8 (40)
	dBP	3 (13)	5 (25)
Mean composite score		**1.16 (1.07)**	**2.40 (1.27)**
Sum of Z-scores		**−2.97 (3.08)**	**2.80 (4.85)**
**Adulthood**			
Neuroendocrine	DHEAS	1 (4)	4 (20)
	Cortisol	7 (28)	7 (35)
Immunological	IGF-1	0 (0)	0 (0)
Metabolic	BMI	4 (16)	8 (40)
	fP-gluc	0 (0)	1 (5)
	Insulin	**0 (0)**	**6 (30)**
	HbA1c	0 (0)	0 (0)
	Cholesterol	4 (17)	2 (10)
	HDL-C	0 (0)	1 (5)
	Triglycerides	0 (0)	0 (0)
Cardiovascular	sBP	2 (8)	5 (25)
	dBP	1 (4)	2 (10)
Mean composite score		**0.72 (0.98)**	**1.85 (1.31)**
Sum of Z-scores		**−2.45 (3.58)**	**1.67 (4.27)**

Values for each biomarker are presented as n (%) of participants whose observed values were considered outside of clinical reference ranges (See Supplemental Table 1 (20) for more detailed information on reference ranges that were used in this study). For each participant the biomarker indicators were summed to calculate mean composite scores, and mean composite score and summed z-scores are expressed as mean (SD). For more information on allostatic load score calculations, see Supplemental Materials. Bolded values indicate statistical significance at the level of *p* < 0.05.

*DHEAS* dehydroepiandrosterone sulfate, *IGF-1* insulin-like growth factor -1, *fP-gluc* fasting glucose, *HDL-C* high-density lipoprotein cholesterol, *BP* blood pressure.

**Fig. 1:**
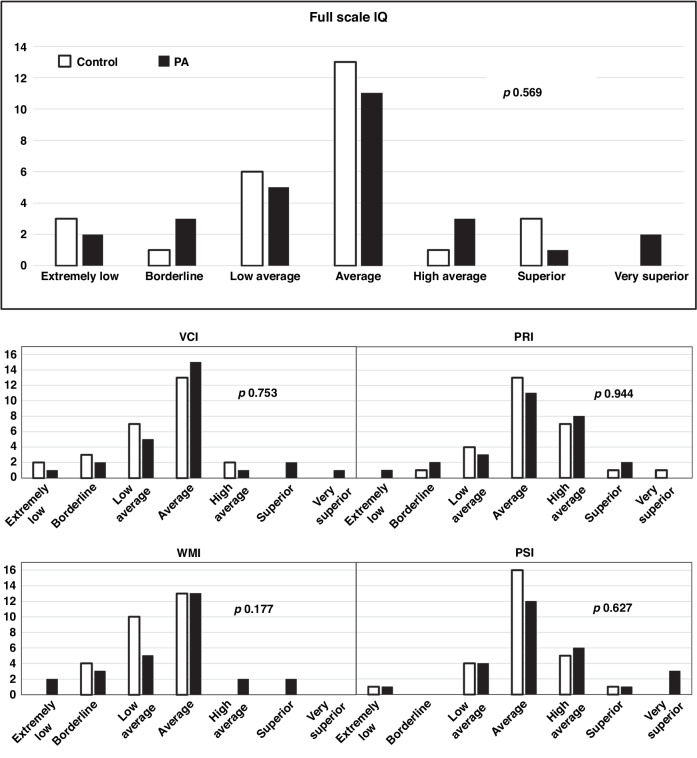
Results from the Wecshler Adult Intelligence Scale (WAIS, fourth edition) assessment in adult women with a history of premature adrenarche (PA) and controls (*n* = 27 for both groups). Bars indicate how many participants belong to each descriptive category. Classifications are as follows: extremely low, <69; borderline, 70–79; low average, 80–89; average, 90–109; high average, 110–119; superior, 120–129; very superior, >130. Differences between the groups were analysed using Fischer´s exact test and *p* values are shown in each panel. IQ intelligence quotient, VCI verbal comprehension index, PRI perceptual reasoning index, WMI working memory index, PSI processing speed index.

There were no significant differences between the groups in mean grades, current occupation and educational levels, or in WAIS-IV assessment results (Table 3). The full-scale IQ and each subsystem score of each participant was also assigned to a descriptive category, and distributions of these classifications were similar between the groups (Fig. 1). Mean comprehensive school education grades correlated positively with FSIQ but not with menarcheal age (Supplemental material, Fig. 4).

**Table 3:** Educational and occupational achievements and the results of the WAIS-IV test in women with a history of premature adrenarche (PA) and controls.

	Control, *n* = 27	PA, *n* = 27	*p*
**Educational and occupational achievements**			
Diploma mean grade^b^	8.1 (1.0)	8.2 (0.8)	0.727^a^
Language skills^c^	8.0 (1.2)	8.3 (1.0)	0.389^a^
Sciences^d^	8.0 (1.1)	8.0 (0.9)	0.922^a^
Social sciences^e^	8.2 (0.9)	8.4 (0.9)	0.489^a^
Current occupation, n (%)			
Student	27 (100)	26 (96)	>0.999
Employee	0 (0)	1 (4)	
Unemployed	0 (0)	0 (0)	
Current school, *n* (%)			
Highschool	17 (63)	13 (50)	0.206
Trade school	10 (37)	9 (35)	
University	0 (0)	3 (11)	
Trade school and high school^f^	0(0)	1 (4)	
**WAIS-IV assessment**			
Full scale IQ	92.7 (16.7)	97.5 (22.5)	0.376
Verbal Comprehension Index Scale	90.2 (14.4)	97.5 (15.7)	0.082
Perceptual Reasoning Index Scale	101.9 (13.3)	100.1 (16.5)	0.543
Working Memory Index Scale	90.0 (9.3)	92.2 (16.0)	0.552
Processing Speed Index Scale	100.0 (13.5)	103.8 (16.0)	0.354
Above average IQ^g^			
Yes	4 (15)	6 (22)	0.484
No	23 (85)	21 (78)	
Below average IQ^h^			
Yes	10 (37)	10 (37)	>0.999
No	17 (63)	17 (63)	

Continuous variables expressed as mean (SD) and analyzed with the t-test. Categorical variables expressed as n (%) and analyzed with the Fischer exact test.

*IQ* intelligence quotient, *WAIS-IV* Wecshler Adult Intelligence Scale (4th ed.).

^a^non-significant also when controlled for locations of the schools.

^b^From comprehensive school in scale of 4–10, including Finnish, English, and Swedish language, math, physics, chemistry, geology, biology, health science, history, religion, and political science.

^c^including Finnish, English, and Swedish.

^d^including math, physics, chemistry, geology, and biology.

^e^including history, religion, and political science.

^f^indicates that the participant is attending both trade school and high school at the same time.

^g^Full-scale IQ > 109.

^h^Full-scale IQ < 90.

In linear regression models (Table 4), only cortisol, but not DHEA(S), glucose, HbA1c, or IGF-1, was a significant predictor for adulthood cognitive outcomes.

**Table 4:** Linear regression models predicting WAIS-IV full-scale intelligence quotient (FSIQ) and different subtest scores, and comprehensive school education diploma mean grades in the 54 adult women.

		FSIQ		VCI		PRI		WMI		PSI		Mean grade^a,b^	
		β	R2	β	R2	β	R2	β	R2	β	R2	β	R2
**Allostatic load**^**c**^													
Composite score 7 years		−0.021	0.088	0.096	0.157	−0.112	0.077	−0.158	0.043	0.071	0.086	−0.202	0.171
Sum of Z-scores 7 years		−0.130	0.137	0.106	0.207	−0.283	0.116	−0.166	0.064	−0.094	0.099	−0.041	0.134
Composite score 18 years		−0.077	0.093	−0.163	0.174	−0.027	0.066	−0.019	0.020	0.001	0.081		
Sum of Z-scores 18 years		0.011	0.094	0.031	0.208	−0.115	0.065	0.038	0.033	0.074	0.122		
**Individual biomarker models**													
Menarcheal age		0.159	0.115	0.134	0.157	0.147	0.063	0.071	0.025	0.132	0.122	−0.153	0.122
Model 1	DHEAS^d^	−0.179	**0.271**	0.037	0.174	−0.075	0.211	−0.267	**0.305**	−0.361	**0.290**	−0.081	0.153
	DHEA^d^	0.011		−0.005		−0.160		0.051		0.217		0.034	
	Cortisol^d^	**−0.385**		−0.071		−0.330		**−0.474**		**−0.429**		−0.161	
Model 2	fP-glucose	−0.025	0.177	−0.053	0.206	−0.023	0.214	−0.022	0.124	0.032	0.130	−0.380	0.289
	HbA1c	−0.280		−0.048		−0.400		−0.226		−0.210		0.139	
	IGF-1	0.033		0.126		−0.153		0.196		−0.012		0.274	

All models are controlled for birth weight SDS, gestational age and current illness. Comprehensive school education mean grade includes all grades except for music, arts, sports and handcraft. Bolded values indicate statistical significance at the level of p < 0.05.

*FSIQ* full-scale intelligence quotient, *VCI* verbal comprehension index, *PRI* perceptual reasoning index, *WMI* working memory index, *PSI* processing speed index, *DHEAS* dehydroepiandrosterone sulfate, *DHEA* dehydroepiandrosterone, *IGF-1* insulin-like growth factor 1.

^a^mean grades were available for 20 PA and 20 control women.

^b^controlled also for the different schools that the participants attended.

^c^for allostatic load evaluations the sample size was 45 (25 controls and 20 PA).

^d^log transformed to parametric distribution.

## Discussion

While no other study has examined cognitive performance in adults with a history of PA, our results suggest that PA does not lead to long-term effects on cognitive performance in girls. In children, one prior study reported lower IQ among a small sample of PA children compared to peers,^15^ but later, a larger study found no such differences.^16^ More recently, a Finnish study showed no difference in executive function assessments between children with and without signs of adrenarche, and no association between adrenarcheal hormone concentrations and executive function assessment scores.^17^ Our results support these latter studies, as neither DHEA(S) nor a history of PA was associated with adulthood cognitive performance. In particular, while some prior studies^6^ have found the increasing levels of DHEA in adolescence, and related changes in brain structure and connectivity to be linked with improved overall working memory,^8^ and visual attention and processing,^7^ childhood DHEA in our study failed to predict WMI, VCI and PRI in adulthood.

Dysfunction of glucose metabolism may affect neurocognitive outcomes in a non-beneficial way. Type 2 diabetes in adolescence has been associated with lower scores in all cognitive domains^25^ and at adult age, even prediabetic IR has been found to negatively correlate with cognitive performance.^26^ A recent study about women with polycystic ovary syndrome (PCOS) found that hyperandrogenic PCOS women with IR had lower executive function domain scores compared to PCOS women without IR.^27^ Although participants in our study were not diabetic and we are not able to define the exact prevalence of PCOS in our cohort, PA women did have increased weight, glucose metabolism disturbances, and hyperandrogenism, yet still scored similarly to controls in all outcome measures. Furthermore, childhood glucose, HbA1c, and IGF-1 failed to predict adulthood outcomes. Despite the fact that in childhood the PA group had higher absolute glucose metabolism biomarker concentrations compared to controls, there were no differences in the proportions of those whose glucose, HbA1c, insulin or IGF-1 exceeded the normal reference (Table 2.). In fact, very few of the whole study population had elevated values. It is thus possible that the discrepancy between our and previous studies may be due to the fact that the deviation in the participants’ glucose metabolism was simply insufficient to provide any marked effects on cognitive performance in the regression models.

In addition to the effects that premature sex steroid exposure in itself might exert on the developing brain,^9,10^ added vulnerability to psychosocial stress from earlier maturation may lead to maladaptive neurodevelopmental trajectories, and thereby to nonbeneficial adulthood outcomes.^9,11^ AL scores, reflecting the wear and tear of the body from repeated and prolonged stressful situations,^23^ were indeed higher in our PA group in both childhood and adulthood, and PA women did go through menarche earlier than controls. These characteristics, however, neither led to nor correlated with adverse consequences in terms of cognitive performance. Some prior studies have found earlier menarche to be linked with poorer academic performance.^11^ Although the correlation between menarcheal age and mean comprehensive school education grade was not significant in our study, it trended towards the opposite effect with higher mean grades in those with earlier menarche. Regarding AL, an earlier longitudinal study found increased AL in adolescence to be linked with impaired working memory in young adulthood,^28^ and another cross-sectional study showed similar results among 20–59-year-old adults,^29^ while our study found no such associations. One possible explanation for the incongruousness between our and previous studies could be that the AL scores of the PA women were elevated due to reasons unrelated to maturational timing and adaptation to stress. Indeed, the association between a history of PA and adulthood AL score (*β* = 0.422, *p* *=* 0.004) was unaltered by menarcheal age adjustment, and PA women in our cohort were previously found to have good health-related quality of life at age 12,^30^ including self-assessed levels of mental function, distress, depression and friendships, suggesting relatively low chance of excessive prolonged stress. Furthermore, differences in childhood and adulthood AL composite scores were driven by single biomarkers (DHEAS and insulin, respectively). Increased AL due to adaptation to prolonged stress should, by definition, be evident across different systems.^23^ It is thus not surprising, that AL scores as such failed to predict adulthood cognitive performance.

The strength of this study lies in its longitudinal format with comprehensive clinical and biochemical data on two time points. However, the sample size is relatively small, and data on confounders such as parental cognitive abilities and socio-economic status are missing. Of note, three controls and two PA individuals scored extremely low on WAIS-IV assessment (IQ < 69), suggestive of intellectual disability. None, however, had such a diagnosis, any other long-term illnesses or used any medication. Two of these five did not receive added support during comprehensive education and still scored surprisingly good grades. It is possible that these five were simply unable to perform to the best of their ability during our WAIS-IV assessment. Either way, removing them from the sample did not change the results (Supplemental Table 2). Selection and attrition biases are possible, as the groups may represent the healthier and socioeconomically more fortunate part of the population, although the data available does not support this (Supplemental Figs. 1, 2). Also, the groups were constituted of Caucasian adolescents from Finland - a western, educated, industrialized, rich and democratic (WEIRD) country. Earlier studies have shown that timing of maturational changes varies between ethnicities^11^ so that PA individuals of different ethnic background may be affected by these changes to a greater degree. And as some aspects of the mechanisms discussed above are intersocial, or even cultural in nature, the results may not be fully generalizable to other, especially non-WEIRD, populations.

In conclusion, our study suggests that girls with PA are not predisposed to long-term changes in cognitive performance. Excessive and premature adrenal androgen exposure and glucose metabolism dysfunction in childhood, and elevated AL scores in childhood and adulthood neither led to nor correlated with any adverse adulthood cognitive outcomes.

## Supplementary information

Supplementary materials

Supplemental Table 3

## Data Availability

The data that support the findings of this study are not publicly available due to their containing information that could compromise the privacy of research participants but are available from the corresponding author [J.T.] upon reasonable request.
