# Supplementary material for: Cognitive performance in young adult women with a history of premature adrenarche
Source: Pediatr Res. 2024 Jul 5;97(2):714–22. doi: 10.1038/s41390-024-03380-4 (PMC12015106; doi:10.1038/s41390-024-03380-4)
Supplement: Supplementary file 1 — Supplementary materials [file 41390_2024_3380_MOESM1_ESM.pdf]

Supplemental materials

Cognitive performance in young adult women with a history of premature adrenarche

Jussi Tennilä[1], Liisa Muukkonen[2], Pauliina Utriainen[3,4], Raimo Voutilainen[1], Jarmo Jääskeläinen[1], Jani Liimatta[1,5]

[1]Kuopio Pediatric Research Unit, University of Eastern Finland and Kuopio University Hospital, 70211 Kuopio, Finland

[2] Coronaria Psykiatria, 80100 Joensuu, Finland

[3]Research Program for Clinical and Molecular Metabolism, Faculty of Medicine, University of Helsinki, 00014 Helsinki, Finland

[4]Pediatric Research Center, Children's Hospital, Helsinki University Hospital, 00029 Helsinki, Finland

[5]Department of BioMedical Research, University of Bern, 3012 Bern, Switzerland

Supplemental Figure 1. Flow chart of the recruitment process.

Flow chart of the recruitment process

	PA (n)	Control (n)
Originally recruited (mean age 7.2 years)	63	80
7 PA girls recruited later	70	
Attended follow-up in adulthood (mean age 18.5 years)	30	42
Willing to perform WAIS-IV	28	29
Excluded due to birth before 35 weeks gestational age	1	2
Total	27	27

Allostatic load calculated for: 20 PA & 25 controls[a]	Comprehensive school diploma mean grade evaluated for: 20 PA & 20 controls[b]	WAIS-IV assessment: 27 PA & 27 controls

	PA (n)	Control (n)
Letter sent to non-attendees	40	38
Responded	12	11

Notes: a, due to missing data in childhood and/or adulthood allostatic load component variables; b, due to missing data, and the fact that three participants had received additional support and possibly modified testing criteria, leading to ineligibility for grade comparisons.

Supplemental Table 1. Reference ranges for calculating allostatic load scores.

Allostatic load component	Reference range	Ref.
**At prepuberty**		
DHEAS, umol/l	<1.08	Guran et al. 2015[1]
Cortisol, nmol/l	113-597	Elmlinger et al. 2002[2]
IGF-1, nmol/l	>8.12	Ertl et al. 2014[3]
ISO-BMI, kg/m^2	18.5-25	Saari et al. 2011[4]
fP-glucose, mmol/l	<5.6	ADA guideline[5]
Insulin, mU/l	<66.23	Peplies 2014[6]
HbA1c, mmol/mol	<39	ADA guideline[5]
Cholesterol, mmol/l	<4.9	AAP guideline[7]
HDL-C, mmol/l	>1.2	
Triglycerides, mmol/l	<1.2	
Systolic BP, mmHg	Age and height specific	AAP guideline[8]
Diastolic BP, mmHg	Age and height specific	
**At adulthood**		
DHEAS, umol/l	<12.76	Bokulic et al. 2023[9]
Cortisol, nmol/l	159-569 / 228-994[a]	Panton et al. 2019[10]
IGF-1, nmol/l	>14.54	Friedrich 2008[11]
BMI, kg/m^2	18.5-25	WHO guideline[12]
fP-glucose, mmol/l	<6	ESC guideline[13]
Insulin, mU/l	<12.64	Tohidi et al. 2014[14]
HbA1c, mmol/mol	<48	ESC guideline[13]
Cholesterol, mmol/l	<5.2	NCEP guideline[15]
HDL-C, mmol/l	>1.0	
Triglycerides, mmol/l	<1.7	
Systolic BP, mmHg	<129	ESC/ESH guideline[16]
Diastolic BP, mmHg	<84	

Notes: a, 159-569 for those who did not use ethinyl estradiol contraceptives, and 228-994 for those who did. Abbreviations: BMI, body mass index; BP, blood pressure; DHEAS, dehydroepiandrosterone sulfate; fP-, fasting plasma; HbA1c, hemoglobin A1c; HDL-C, high-density lipoprotein cholesterol; IGF-1, insulin-like growth factor 1.

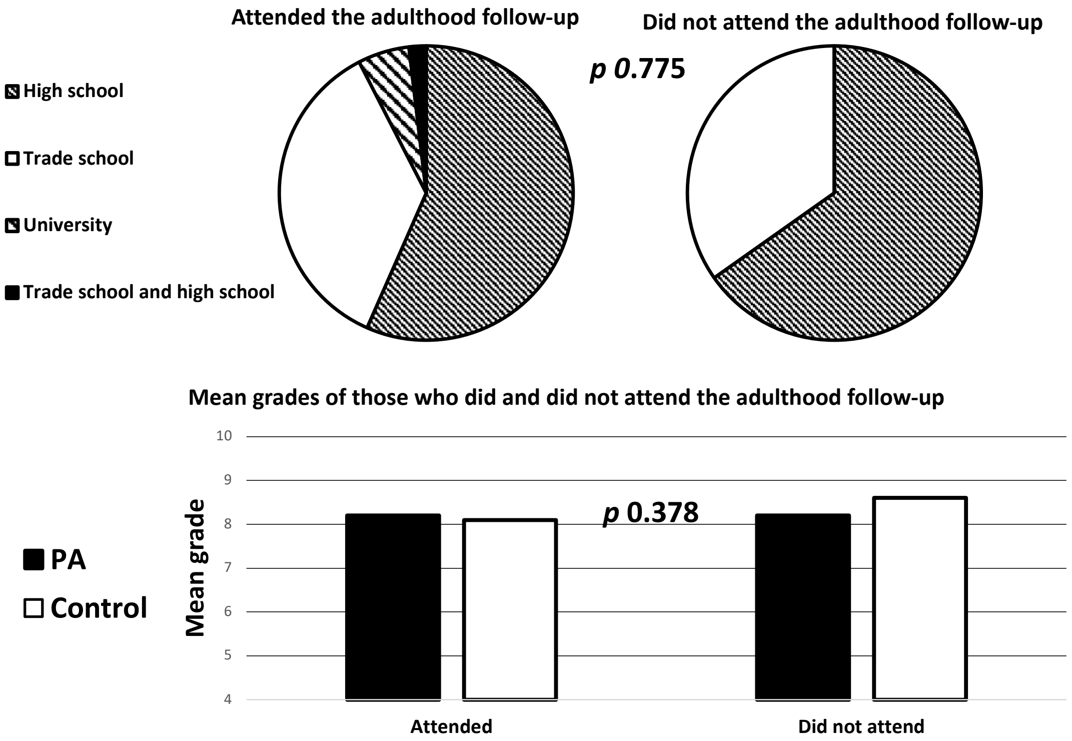

Supplemental Figure 2. Current schools (upper panel) and mean grades from primary school (lower panel) in those who attended the adulthood follow-up and those who did not. This information for those who did not attend the adulthood follow-up was gathered with a questionnaire sent to the non-attendees. Tightly dashed section in upper panel depicts proportions for those in high school; white section, trade school; sparsely dashed, university and high school simultaneously; black, trade school and high school simultaneously. Black and white bars in the lower panel indicate mean grades in individuals with and without a history of premature adrenarche (PA), respectively. Differences between those who attended and who did not attend the adulthood follow-up are analyzed using either the Fischer´s exact test (upper panel) or t-test (lower panel), and p-values are shown in each panel.

Supplemental Table 2. Outcome measures for the 24 controls and 25 PA participants with full-scale IQ above 69.

	Control, n = 24	PA, n = 25	*p*
**Educational and occupational achievements**			
Diploma mean grade[b]	8.3 (0.9)	8.1 (0.9)	0.557[a]
Language skills[c]	8.2 (1.0)	8.2 (1.0)	0.955[a]
Sciences[d]	8.2 (1.0)	7.9 (1.0)	0.305[a]
Social sciences[e]	8.2 (1.0)	8.3 (1.0)	0.878[a]
Current occupation, n (%)			
Student	24 (100)	24 (96)	
Employee	0 (0)	1 (4)	>0.999
Unemployed	0 (0)	0 (0)	
Current school, n (%)			
High school	17 (71)	13 (54)	
Trade school	7 (29)	7 (29)	0.229
University	0 (0)	3 (13)	
Trade school and high school[f]	0(0)	1 (4)	
**WAIS-IV assessment**			
Full scale IQ	96.5 (13.1)	101.4 (18.2)	0.292
Verbal Comprehension Index Scale	92.2 (13.8)	99.5 (14.4)	0.074
Perceptual Reasoning Index Scale	104.8 (11.2)	103.0 (12.9)	0.624
Working Memory Index Scale	91.9 (8.0)	94.6 (14.1)	0.414
Processing Speed Index Scale	102.3 (11.5)	105.6 (14.4)	0.380
Above average IQ[g]			
Yes	4 (17)	6 (24)	0.725
No	20 (83)	19 (76)	
Below average IQ[h]			
Yes	7 (29)	8 (32)	>0.999
No	17 (71)	17 (68)	

Continuous variables expressed as mean (SD) and analyzed with the t-test. Categorical variables expressed as n (%) and analyzed with the Fischer exact test.

Notes: a, non-significant also when controlled for locations of the schools; b, From comprehensive school in scale of 4-10, including Finnish, English and Swedish language, math, physics, chemistry, geology, biology, health science, history, religion, and political science; c, including Finnish, English and Swedish; d, including math, physics, chemistry, geology, and biology; e, including history, religion, and political science; f, indicates that the participant is attending both trade school and high school at the same time; g, Full-scale IQ > 109; h, Full-scale IQ < 90.
Abbreviations: IQ, intelligence quotient; WAIS-IV; Wecshler Adult Intelligence Scale (4th ed.).

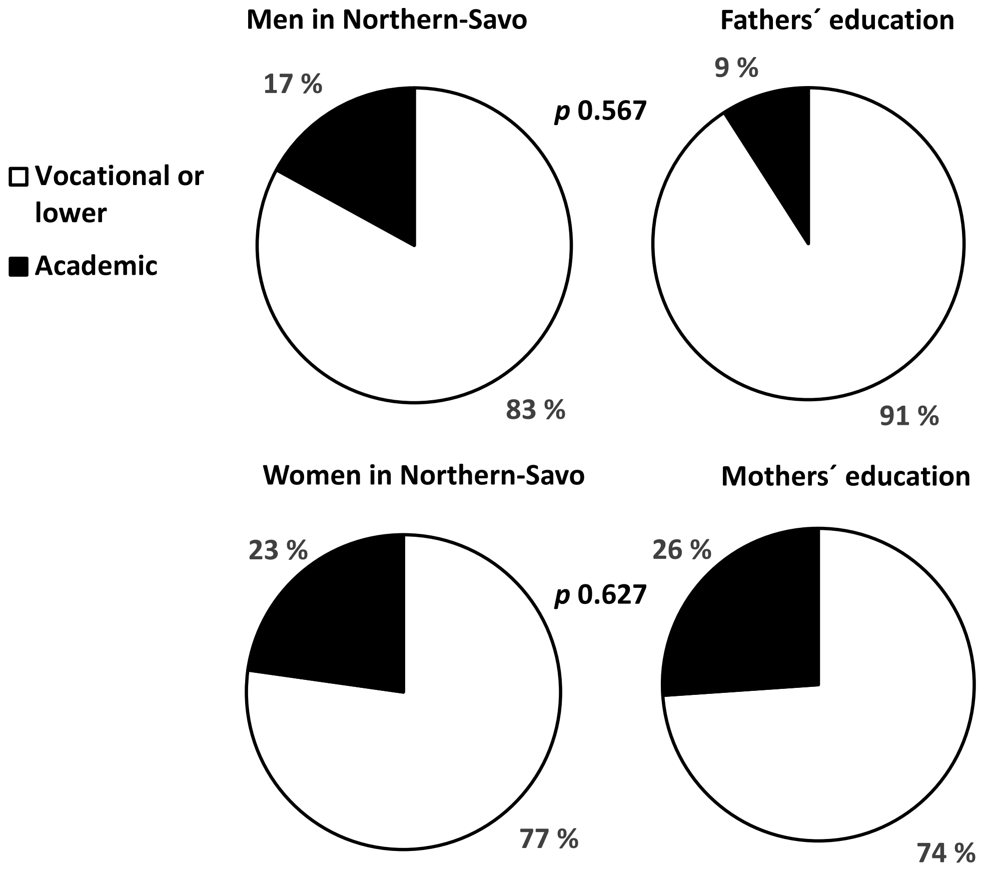

Supplemental Figure 3. Proportions of educational status (academic vs. vocational or lower) among general adult population in Northern Savo region and among parents of those who did not attend the adulthood follow-up. “Academic” refers to a degree from a university (bachelors, masters or doctoral degree), and “Vocational or lower” includes all other educational levels. Educational levels among 35–69-year-old men and women in Northern-Savo region in 2016 were obtained from Official Statistics of Finland[17]. Differences between the reference population and either fathers (upper panels) or mothers (lower panels) of individuals who did not attend adulthood follow-up were analyzed using the Fischer´s exact test and p-values are shown.

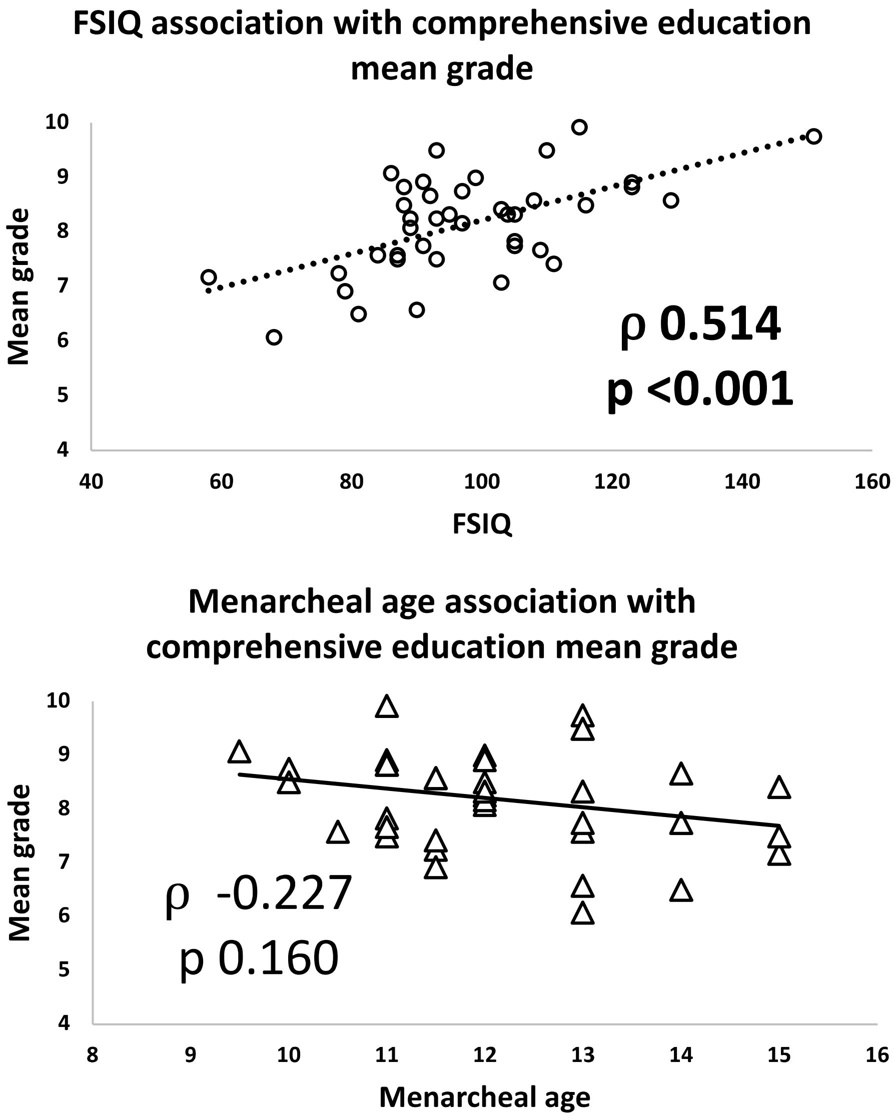

Supplemental Figure 4. Relationships between primary school mean grades and full-scale intelligence quotient (FSIQ; upper panel) or menarcheal age (lower panel). Grades are on a 7-ladder scale with 10 being the highest grade and 4 meaning failed. In Finland teenagers graduate from primary school at approximately 14-15 years of age. Sample size for these analyses was 40. Values indicate Spearman´s rho correlation coefficients and p-values.

Supplemental method description for calculations of allostatic load (AL)

A total of 12 biomarkers were collected from the participants in preadolescence and again at adult age. The following subsystems and corresponding biomarkers were examined: neuroendocrine system, DHEAS and cortisol; immunological system, IGF-1; metabolic system, BMI, glucose, insulin, HbA1c, total cholesterol, high-density lipoprotein cholesterol (HDL-C), triglycerides; cardiovascular system, systolic blood pressure (sBP), diastolic blood pressure (dBP).

Multiple different algorithms have been used to calculate AL scores[18]. Preadolescent PA individuals, by definition, differ from peers in some biomarker concentrations, such as DHEAS. Therefore, we opted for the clinical cut-off algorithm[19]. For each biomarker, the relevant clinical cut-off reference ranges were identified, and a dichotomous variable was created indicating whether each particular value was within reference ranges considered normal or low risk (coded "0"), or outside of said ranges, thereby considered high risk (coded "1"). Where no clinically relevant cut-off point could be identified, we used established reference ranges and picked most suitable percentiles according to prior knowledge. When possible, we used different reference ranges for those who used ethinyl estradiol contraception and those who did not (e.g. for cortisol). Table 1. depicts these cut-off points and corresponding references. If a participant used a relevant medication, then that/those particular biomarkers were coded "1".

Next, missing variables were handled. AL scores were only calculated if more than half of the 12 variables in childhood, and more than half of the variables in each subsystem in adulthood were present. This procedure resulted in 7 PA and 2 control women being ineligible for AL

score calculation due to missing data, so that ultimately the scores were calculated for 20 PA and 25 controls.

At childhood phase the AL scores were simply the sum of all individual indicators (Range 0-12; expressed as mean (SD)), without calculating subsystem scores. This method is considered most appropriate for childhood AL score measurement[20]. To account for the rest of the missing variables at childhood stage, the present variables were first summed, and this sum was then multiplied by (12 / (no. of variables present)). This was not a major ordeal, as all but two (0.04%) of the total 540 values were present.

In adulthood, each indicator variable was first summed to form subsystem scores (neuroendocrine, immunological, metabolic and cardiovascular). If a participant was missing more than half of the variables in any individual subsystem, the AL score was not calculated. Rest of the missing variables were imputed by summing present variables into subsystem scores, and then multiplying that value by ((no. of variables in the subsystem) / (no. of variables present)). 8 values out of a total of 540 (1.5%) were missing and so imputed at this stage. These subsystem scores were then summed to create the AL composite score (range 0-12, expressed as mean (SD)).

In addition, to see if a different approach would change the results, we calculated z-scores for each variable with the following formula: (observed value - sample mean) / sample SD. Before calculating z-scores the following variables were log transformed to normalize distributions: prepubertal DHEAS, cortisol, ISO-BMI, triglycerides, and insulin; adulthood DHEAS, cortisol, IGF-1, BMI, fasting glucose, insulin, total cholesterol and triglycerides. For each participant the Z-scores were then summed creating another commonly used AL score – the sum of z-scores.

References

1. Guran, T. et al. Reference values for serum dehydroepiandrosterone-sulphate in healthy children and adolescents with emphasis on the age of adrenarche and pubarche. *Clin. Endocrinol. (Oxf).* **82**, 712-8 (2015).

2. Elmlinger, M. W., Kühnel, W. & Ranke M. B. Reference ranges for serum concentrations of lutropin (LH), follitropin (FSH), estradiol (E2), prolactin, progesterone, sex hormone-binding globulin (SHBG), dehydroepiandrosterone sulfate (DHEAS), cortisol and ferritin in neonates, children and young adults. *Clin. Chem. Lab. Med*. **40**, 1151-60 (2002).

3. Ertl, D. A., Gleiss, A., Sagmeister, S. & Haeusler G. Determining the normal range for IGF-I, IGFBP-3, and ALS: new reference data based on current internal standards. *Wien. Med. Wochenschr.* **164**, 343-52 (2014).

4. Saari, A. et al. New Finnish growth references for children and adolescents aged 0 to 20 years: Length/height-for-age, weight-for-length/height, and body mass index-for-age. *Ann. Med*. **43**, 235-48 (2011).

5. Arslanian S, et al. Evaluation and management of youth-onset type 2 diabetes: A position statement by the American diabetes association. *Diabetes Care.* **41** 2648-68 (2018)

6. Peplies J, et al. IDEFICS consortium. Percentiles of fasting serum insulin, glucose, HbA1c and HOMA-IR in pre-pubertal normal weight European children from the IDEFICS cohort. *Int. J. Obes. (Lond).* **38**, S39-47 (2014).

7. Daniels, S. R. & Greer, F. R. Committee on Nutrition. Lipid screening and cardiovascular health in childhood. *Pediatrics.* **122**, 198-208 (2008).

8. Flynn JT, et al. Subcommittee on screening and management of high blood Pressure in children. Clinical Practice Guideline for Screening and Management of High Blood Pressure

in Children and Adolescents. *Pediatrics.* 2017; 140(3): e20171904 (2017). Erratum in: *Pediatrics.* Nov 30 (2017). Erratum in: *Pediatrics.* **142**, e20181739 (2018)

9. Bokulic, A., Zec, I., Marijancevic, D. & Goreta, S. Androgens in women: Establishing reference intervals for dehydroepiandrostenedione sulphate and androstenedione on the Roche Cobas. *Biochem. Med. (Zagreb).* **33**, 020706 (2023).

10. Panton, K.K. et al. New reference intervals for cortisol, cortisol binding globulin and free cortisol index in women using ethinyl estradiol. *Scand. Clin. J. Lab. Invest.* **79**, 314-9 (2019).

11. Friedrich N, et al. Reference ranges of serum IGF-1 and IGFBP-3 levels in a general adult population: results of the Study of Health in Pomerania (SHIP). *Growth Horm. IGF Res.* 2008; **18**, 228-37 (2008).

12. Obesity: preventing and managing the global epidemic. Report of a WHO consultation. **894**: 1-253 (2000).

13. Cosentino, F. et al. ESC Scientific Document Group. 2019 ESC Guidelines on diabetes, pre-diabetes, and cardiovascular diseases developed in collaboration with the EASD. *Eur. Heart. J*. 2020 Jan 7; **41(2)**, 255-323. Erratum in: *Eur Heart J.* **41(45)**, 4317 (2020).

14. Tohidi, M. et al. Age- and sex-specific reference values for fasting serum insulin levels and insulin resistance/sensitivity indices in healthy Iranian adults: Tehran Lipid and Glucose Study. *Clin. Biochem*. **47**, 432-8 (2014).

15. Expert Panel on Detection, Evaluation, and Treatment of High Blood Cholesterol in Adults. Executive Summary of The Third Report of The National Cholesterol Education Program (NCEP) Expert Panel on Detection, Evaluation, And Treatment of High Blood Cholesterol In Adults (Adult Treatment Panel III). *JAMA*. **285**, 2486-97 (2001).

16. Williams, B. et al. ESC Scientific Document Group. 2018 ESC/ESH Guidelines for the management of arterial hypertension. *Eur. Heart. J.* **39**, 3021-104 (2018). Erratum in: *Eur. Heart. J.* **40**, 475 (2019).

17. Official Statistics of Finland (Internet). ISSN=1799-4586. Helsinki: Educational Structure of Population: Population aged 15 or over by level of education, municipality, gender and age, 1970-2021. (Accessed June 2nd 2023). Available from: https://pxdata.stat.fi/PxWeb/pxweb/en/StatFin/StatFin__vkour/statfin_vkour_pxt_12bq.px/

18. Juster, R. P., Mcewen, B. S. & Lupien, S. J. Allostatic load biomarkers of chronic stress and impact on health and cognition. *Neurosci. Biobehav. Rev.* **35**, 2-16 (2010).

19. Seeman, T. et al. Education, income and ethnic differences in cumulative biological risk profiles in a national sample of US adults: NHANES III (1988-1994). *Soc. Sci. Med.* **66**, 72-87 (2008).

20. King, A. L., Garnier-Villareal, M., Simanek, A. M. & Johnson, N. L. Testing allostatic load factor structures among adolescents: A structural equation modeling approach. *Am. J. Hum. Biol.* **31**, e23242 (2019).
